# Safety and prognostic value of regadenoson stress cardiovascular magnetic resonance imaging in heart transplant recipients

**DOI:** 10.1186/s12968-018-0515-2

**Published:** 2019-01-24

**Authors:** Felipe Kazmirczak, Prabhjot S. Nijjar, Lei Zhang, Andrew Hughes, Ko-Hsuan Amy Chen, Osama Okasha, Cindy M. Martin, Mehmet Akçakaya, Afshin Farzaneh-Far, Chetan Shenoy

**Affiliations:** 1 Cardiovascular Division, Department of Medicine, University of Minnesota Medical Center, 420 Delaware Street SE, MMC 508, Minneapolis, MN 55455 USA; 2 Clinical and Translational Science Institute, University of Minnesota, Minneapolis, MN USA; 3 University of Minnesota Medical School, Minneapolis, MN USA; 4 Department of Electrical and Computer Engineering and Center for Magnetic Resonance Research, University of Minnesota, Minneapolis, MN USA; 5 Division of Cardiology, Department of Medicine, University of Illinois at Chicago, Chicago, IL USA

**Keywords:** Cardiovascular magnetic resonance, Stress perfusion, Vasodilator, Regadenoson, Safety

## Abstract

**Background:**

There is a critical need for non-invasive methods to detect coronary allograft vasculopathy and to risk stratify heart transplant recipients. Vasodilator stress testing using cardiovascular magnetic resonance imaging (CMR) is a promising technique for this purpose. We aimed to evaluate the safety and the prognostic value of regadenoson stress CMR in heart transplant recipients.

**Methods:**

To evaluate the safety, we assessed adverse effects in a retrospective matched cohort study of consecutive heart transplant recipients who underwent regadenoson stress CMR matched in a 2:1 ratio to age- and gender-matched non-heart transplant patients. To evaluate the prognostic value, we compared the outcomes of patients with abnormal vs. normal regadenoson stress CMRs using a composite endpoint of myocardial infarction, percutaneous intervention, cardiac hospitalization, retransplantation or death.

**Results:**

For the safety analysis, 234 regadenoson stress CMR studies were included - 78 performed in 57 heart transplant recipients and 156 performed in non-heart transplant patients. Those in heart transplant recipients were performed at a median of 2.74 years after transplantation. Thirty-four (44%) CMR studies were performed in the first two years after heart transplantation. There were no differences in the rates of adverse effects between heart transplant recipients and non-heart transplant patients. To study the prognostic value of regadenoson stress CMRs, 20 heart transplant recipients with abnormal regadenoson stress CMRs were compared to 37 with normal regadenoson stress CMRs. An abnormal regadenoson stress CMR was associated with a significantly higher incidence of the composite endpoint compared with a normal regadenoson stress CMR (3-year cumulative incidence estimates of 32.1% vs. 12.7%, *p* = 0.034).

**Conclusions:**

Regadenoson stress CMR is safe and well tolerated in heart transplant recipients, with no incidence of sinus node dysfunction or high-degree atrioventricular block, including in the first two years after heart transplantation. An abnormal regadenoson stress CMR identifies heart transplant recipients at a higher risk for major adverse cardiovascular events.

## Background

Heart transplantation is a proven life-saving treatment option for patients with end-stage heart failure. Despite many advances in the field, graft failure and cardiac allograft vasculopathy (CAV) continue to represent significant causes of morbidity and mortality [1]. Early detection of CAV may allow changes in medical therapy and prevention of progression. However, early diagnosis is challenging due to either absent or atypical symptoms related to allograft denervation [2, 3]. This has necessitated routine screening of heart transplant recipients for CAV. Current preferred methods for surveillance involve coronary angiography and intravascular ultrasound. However, these methods expose patients to the risks of an invasive procedure and involves the use of iodinated contrast with its associated risk of kidney injury. This is a particularly important issue since chronic kidney disease is common in these patients. Thus, there is a critical need for noninvasive techniques to assess cardiac allograft function and to detect treatable CAV [4, 5].

Vasodilator stress testing by cardiovascular magnetic resonance imaging (CMR) is a promising technique for the detection of CAV and for the risk stratification of heart transplant recipients. The use of this technique has been limited because adenosine has been associated with super-sensitivity of the denervated sinus and atrioventricular nodes after heart transplantation, resulting in exaggerated sinus node and atrioventricular node suppression [6, 7].

Regadenoson is a newer selective A_2A_ adenosine receptor agonist used for vasodilator stress testing. Studies in non-transplant patients have shown no incidence of high-degree atrioventricular block with regadenoson [8–10] in contrast to a 4–5% incidence with a six-minute infusion of adenosine [11]. This raises the possibility that regadenoson may be safe in heart transplant recipients. However, heart transplant recipients were excluded from all pre-approval studies of regadenoson [8, 12, 13]. Thus, there are a paucity of data on the safety and tolerability of regadenoson in heart transplant recipients. There are also no data on the safety of regadenoson early after transplantation, or on its prognostic value in heart transplant recipients. Accordingly, we aimed to evaluate the safety and the prognostic value of regadenoson stress CMR in heart transplant recipients.

## Methods

### Patients

We identified consecutive heart transplant recipients undergoing regadenoson stress CMR performed on at the University of Minnesota Medical Center, Minneapolis, Minnesota, USA between April 2012 and December 2017. For the safety analysis, these regadenoson stress CMRs were matched by patient age and gender in a 2:1 ratio to comparison regadenoson stress CMRs performed in non-heart transplant patients during the same time period. All stress CMRs were identified from the University of Minnesota Cardiovascular Magnetic Resonance Registry, an ongoing observational registry including all patients that undergo CMR at the University of Minnesota [14]. This study was approved by University of Minnesota’s Institutional Review Board with a waiver of informed consent.

### CMR protocol, and assessment of symptoms and adverse effects

Regadenoson stress CMRs were performed using a 1.5 T scanner (Siemens Aera, Siemens Healthineers, Erlangen, Germany) with phased-array coil systems. All patients underwent a CMR protocol consisting of: 1) cine CMR at rest for assessment of left ventricular (LV) function; 2) gadolinium first pass perfusion imaging 1–2 min after regadenoson injection for assessment of stress perfusion; 3) gadolinium first-pass perfusion imaging without regadenoson for assessment of rest perfusion; and 4) late gadolinium enhancement (LGE) CMR 10–15 min later. Typically, the procedure was completed in 45 min. Patients were asked to refrain from caffeine for 24 h before the regadenoson stress CMR. Patients with pre-existing second- or third-degree atrioventricular block or sinus node dysfunction were excluded. After cine CMR, the patient table was partially pulled outside the scanner bore to allow access to the patient for regadenoson administration. Regadenoson 0.4 mg (Astellas, Northbrook, Illinois, USA) was injected over approximately 10 s into a peripheral vein followed by a 5 mL saline flush. The patient was centered back into the scanner and the perfusion sequence was started within 1–2 min of regadenoson injection. Gadolinium-based contrast (0.075 mmol/kg gadobenate dimeglumine, Bracco Imaging or 0.1 mmol/kg gadobutrol, Bayer HealthCare LLC, Milan, Italy) was infused at 4–5 ml/s followed by a saline flush (50 ml) via an antecubital vein for both stress and rest perfusion. All patients were monitored by CMR-compatible, three-lead wireless continuous electrocardiogram (ECG) system and pulse oximetry during the study. Blood pressure was monitored before and after regadenoson administration, and a 12-lead ECG was performed before and after the study. Prior to April 2014, aminophylline was used for significant patient symptoms. All studies performed after April 2014 routinely received aminophylline 100 mg intravenously for reversal of hyperemia after stress images were acquired [15]. Patients were routinely asked about their symptoms before and after regadenoson and aminophylline administration. Stress-related adverse events including death, myocardial infarction, ventricular tachycardia, ventricular fibrillation, hospitalization, bronchospasm, and non-life-threatening arrhythmias were noted in the electronic medical record by trained nurses. For this study, data on arrhythmias were extracted from the ECGs and the nurse notes.

### CMR analyses

All stress CMR exams were interpreted blinded to patient outcomes by a consensus of two CMR physicians with > 10 (C. S.) and 2 (F. K.) years of experience respectively. Perfusion and LGE images were assessed in a qualitative fashion. A perfusion defect was identified as a regional dark area that: 1) persisted for > 2 beats while other regions enhanced during the first-pass of contrast through the LV myocardium; and 2) involved the subendocardium. LV systolic dysfunction was defined as an abnormality in global or regional systolic function on cine imaging. Ischemia was defined as a segmental stress perfusion defect without matching hyperenhancement (same location and size) on LGE imaging. A match between a perfusion defect and hyperenhancement on LGE was considered as fibrosis without ischemia. As in routine clinical practice, systolic dysfunction, ischemia and LGE images were interpreted in a binary fashion as normal or abnormal. A normal regadenoson stress CMR was defined as normal global and regional LV systolic function, no ischemia and no fibrosis.

### Assessment of clinical outcomes

Follow up data were collected through review of patient medical records from all locations within our institution’s health system. Mortality status and death dates were also cross-referenced with data from the Minnesota Department of Health’s Office of Vital Records. Collected outcomes included: myocardial infarction, percutaneous intervention, cardiac hospitalization, retransplantation and death. These events together formed the composite endpoint of major adverse cardiovascular events.

### Statistics

Continuous variables were expressed as means and standard deviations, or medians and inter-quartile ranges (IQR) for data that were not normally distributed. Regadenoson stress CMR in the heart transplant recipient and comparison groups were compared using generalized estimating equations in the form of linear regression or logistic regression, as appropriate. Prognostic endpoints were compared using Kaplan-Meier survival analyses and log rank testing. All tests were two-tailed. A p of < 0.05 was used to denote statistical significance. Analyses were performed using SAS version 9.4 (SAS Institute, Cary, North Carolina, USA).

## Results

### Patients

In the study group, 78 regadenoson stress CMRs were performed in 57 unique heart transplant recipients at a median of 2.74 (interquartile range 1.02–7.25) years after transplantation. Thirty-four (44%) CMRs were performed in the first two years after heart transplantation.

Forty-one patients had one, 12 patients had two, three patients had three, and one patient had four regadenoson stress CMRs each. In the comparison group, only one instance of regadenoson stress CMR was included per patient. Patient characteristics are summarized in Table 1. Compared to the heart transplant recipient group, the comparison group had higher rates hyperlipidemia, tobacco use, prior myocardial infarction, and chronic obstructive lung disease.

**Table 1:** Patient characteristics

Patient characteristic	Heart transplant recipients (*n* = 78)	Non-transplant patients (*n* = 156)	*p* value
Age, years (median, IQR)	50.1 (30.5–61.2)	50.1 (30.9–61.2)	0.01
Male, n (%)	30 (38)	60 (38)	1.00
Body mass index, kg/m2 (median, IQR)	29.0 (24.1–31.9)	29.1 (23.8–34.2)	0.10
Graft age, years (median, IQR)	2.74 (1.02–7.25)	N/A	N/A
Rejection, Grade > 3 ever, n (%)	19 (24.4)	N/A	N/A
Hypertension, n (%)	42 (54)	80 (51)	0.67
Diabetes mellitus, n (%)	19 (24)	27 (17)	0.20
Hyperlipidemia, n (%)	75 (96)	67 (43)	< 0.001
Current tobacco use, n (%)	1 (1)	16 (10)	0.003
Myocardial infarction^a^, n (%)	3 (4)	25 (16)	0.004
Percutaneous intervention^a^, n (%)	9 (12)	19 (12)	0.89
Coronary artery bypass graft^a^, n (%)	0	8 (5)	0.06
Atrial fibrillation^a^, n (%)	5 (6)	8 (5)	0.71
Cerebrovascular accident, n (%)	10 (13)	10 (6)	0.09
Chronic obstructive lung disease, n (%)	6 (8)	29 (19)	0.02
Serum creatinine, mg/dL (± SD)	1.12 (0.36)	0.87 (0.20)	< 0.001
Medications			
Angiotensin converting enzyme-inhibitor, n (%)	15 (19)	46 (29)	0.06
Angiotensin receptor blocker, n (%)	17 (22)	21 (13)	0.14
Beta-blocker, n (%)	10 (13)	65 (42)	< 0.001
Calcium channel blocker, n (%)	18 (23)	19 (12)	0.04
Aspirin, n (%)	72 (92)	59 (38)	< 0.001
ADP/P2Y12 inhibitor, n (%)	7 (9)	13 (8)	0.87
Statin, n (%)	72 (92)	53 (34)	< 0.001
Glucocorticoid, n (%)	14 (18)	N/A	N/A
Purine inhibitor, n (%)	72 (92)	N/A	N/A
Calcineurin inhibitor, n (%)	66 (85)	N/A	N/A
mTOR inhibitor, n (%)	18 (23)	N/A	N/A

*IQR* Interquartile Range, *SD* Standard Deviation; ^a^denotes events after heart transplantation in the heart transplant group

### Baseline ECG characteristics

Baseline electrocardiographic (ECG) characteristics are listed in Table 2. In the heart transplant recipient group, there were no instances of patients with pre-existing sinus node dysfunction or atrioventricular block of any degree. In 24 (31%) instances, patients had a right bundle branch block, which was significantly higher than in the comparison group. In 47 (60%) instances, they had ST-T abnormalities.

**Table 2:** Baseline ECG findings

ECG finding	Heart transplant recipients (*n* = 78)	Non-transplant patients (*n* = 156)	*p* value
Sinus rhythm with no abnormalities, n (%)	19 (24)	49 (31)	0.26
First degree atrioventricular block, n (%)	0	6 (4)	0.18
Left bundle branch block, n (%)	0	0	N/A
Right bundle branch block, n (%)	24 (31)	8 (5)	< 0.001
Atrial fibrillation/flutter, n (%)	1 (1)	4 (3)	0.48
Premature atrial complexes, n (%)	1 (1)	4 (3)	0.48
Premature ventricular complexes, n (%)	1 (1)	9 (6)	0.07
ST-T abnormalities, n (%)	47 (60)	104 (67)	0.33

### Hemodynamic changes

In the heart transplant recipient group, the mean heart rate increased from 92 ± 11 bpm to 107 ± 12 bpm, while it increased from 73 ± 15 bpm to 100 ± 13 bpm in the comparison group. There were no significant changes in pre- and post-stress blood pressures in both the heart transplant recipient and comparison groups (Table 3).

**Table 3:** Hemodynamic findings

Hemodynamic finding	Heart transplant recipients (*n* = 78)	Non-transplant patients (*n* = 156)	*p* value
Pre-stress heart rate, bpm (± SD)	92 (11)	73 (15)	< 0.001
Pre-stress systolic blood pressure, mm Hg (± SD)	124 (18)	127 (19)	0.27
Pre-stress diastolic blood pressure, mm Hg (± SD)	80 (13)	78 (13)	0.21
Peak heart rate, bpm (± SD)	107 (12)	100 (13)	< 0.001
Post-stress heart rate, bpm (± SD)	95 (12)	74 (16)	< 0.001
Post-stress systolic blood pressure, mm Hg (± SD)	124 (21)	126 (19)	0.64
Post-stress diastolic blood pressure, mm Hg (± SD)	81 (13)	76 (13)	0.01

*SD* Standard Deviation

### Adverse effects

All regadenoson stress CMRs were completed in both heart transplant recipient and comparison groups. Adverse effects are listed in Table 4. One stress CMR in a heart transplant recipient had to be temporarily interrupted due to regadenoson-related abdominal cramps; the patient received a second dose of regadenoson after 20 min without any further symptoms. Side-effects requiring an intervention occurred in two patients (3%) in the heart transplant recipient group – one had chest pain requiring nitroglycerin and one had symptomatic hypotension requiring intravenous fluids – and in one patient (0.6%) in the comparison group that had symptomatic hypotension requiring intravenous fluids (*p* = 0.26). In all three cases, the symptoms resolved with treatment and the patients were discharged home after the regadenoson stress CMR. Minor side-effects not requiring any interventions such as dyspnea, nausea and headache occurred at similar rates in both groups. There were no occurrences of death, asystole, sinus pause, sinus arrest, high-degree atrioventricular block, ventricular arrhythmias, stress-induced atrial fibrillation, or myocardial infarction. No patients required hospitalization or emergency room evaluation.

**Table 4:** Adverse effects

Adverse effect	Heart transplant recipients (*n* = 78)	Non-transplant patients (*n* = 156)	*p* value
Death, n (%)	0	0	N/A
Asystole, n (%)	0	0	N/A
Sinus pause or arrest, n (%)	0	0	N/A
High-grade atrioventricular block, n (%)	0	0	N/A
Ventricular tachycardia or ventricular fibrillation, n (%)	0	0	N/A
Atrial fibrillation, n (%)	0	0	N/A
Chest pain requiring sublingual nitroglycerin, n (%)	1 (1)	0	0.33
Myocardial infarction, n (%)	0	0	N/A
Symptomatic hypotension, n (%)	1 (1)	1 (0.6)	0.65
Dyspnea, n (%)	6 (7)	9 (6)	0.58
Nausea, n (%)	6 (7)	3 (2)	0.08
Headache, n (%)	2 (3)	0	0.11
Allergic reaction (rash, hives, etc.), n (%)	0	0	N/A
Contrast extravasation, n (%)	0	2 (1)	0.55
Thrombophlebitis, n (%)	0	0	N/A
Hospitalization, n (%)	0	0	N/A

### Clinical outcomes

**Fig. 1:**
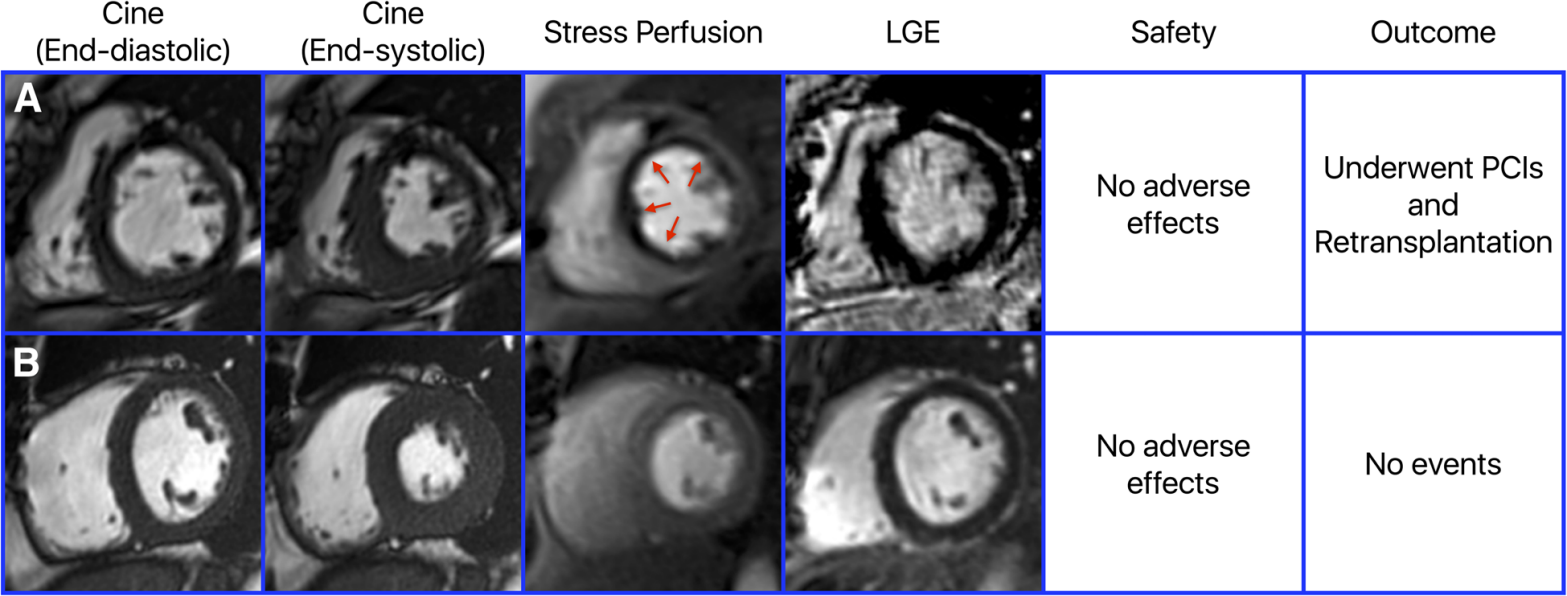
Example images of heart transplant recipients from the study. Panel **a** shows a patient with a decreased left ventricular (LV) ejection fraction of 41% and ischemia in multiple coronary artery territories without late gadolinium enhancement. The patient had no adverse effects from the stress CMR and on follow-up underwent multiple percutaneous interventions and eventually a retransplantation. Panel **b** shows a patient with a normal LV ejection fraction of 55%, no ischemia and no late gadolinium enhancement. The patient had no adverse effects from the stress CMR and on follow-up had no events

**Fig. 2:**
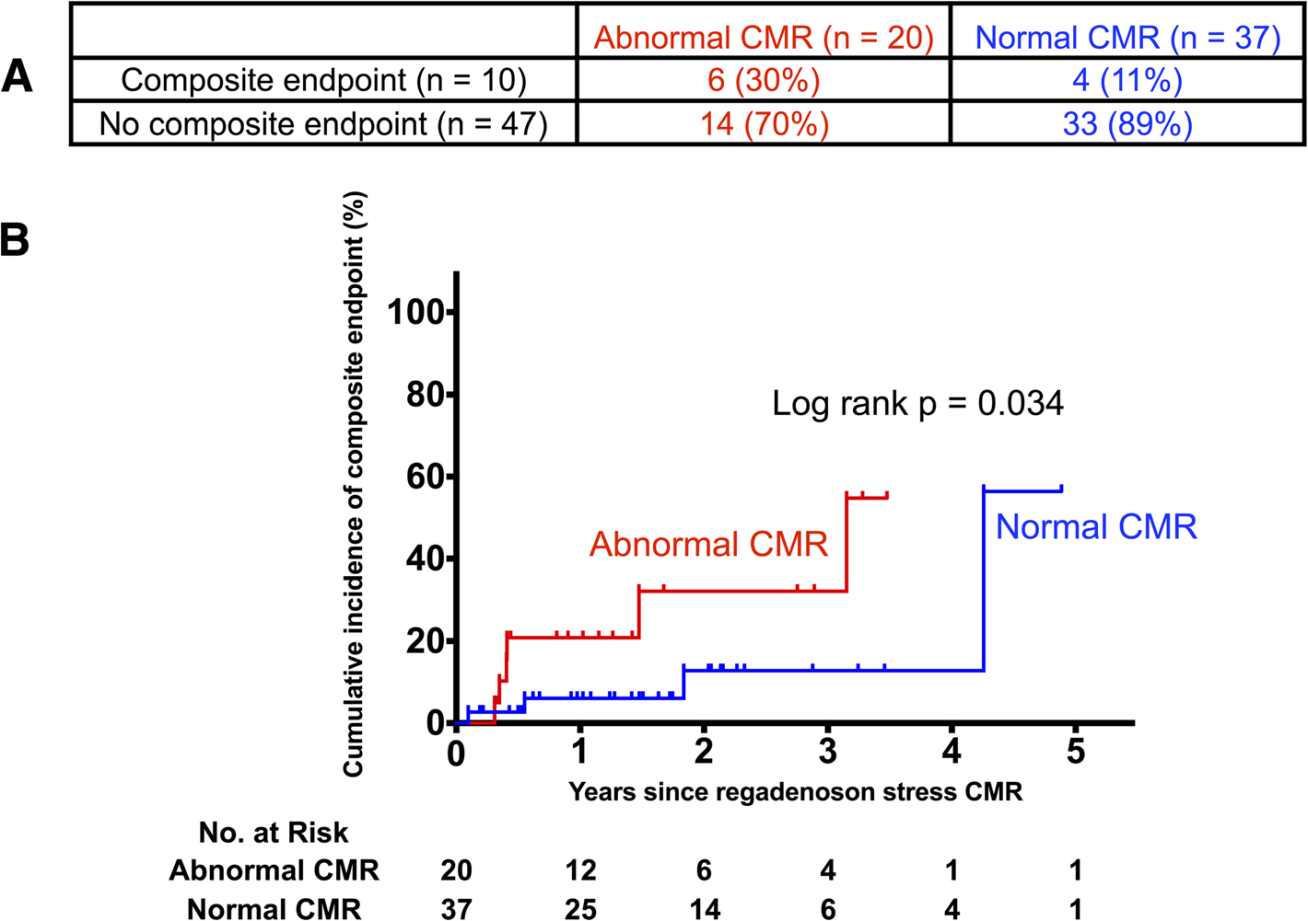
Incidence of composite endpoints according to abnormal vs. normal regadenoson stress CMRs. Panel **a** outlines the rates of the composite endpoint according to abnormal vs. normal regadenoson stress CMRs. Kaplan-Meier curves in Panel **b** demonstrate that cumulative incidence estimate of the composite endpoint is significantly higher in patients with abnormal vs. normal regadenoson stress CMRs

Among 57 heart transplant recipients, 20 had an abnormal regadenoson stress CMRs while 37 had normal testing. Example images of heart transplant recipients from the study are provided in Fig. 1. At a median follow up of 1.3 years (interquartile range 0.5–2.1 years), there were no instances of myocardial infarction, four percutaneous coronary interventions, four cardiac hospitalizations, three retransplantations, and four deaths, accounting for 10 composite outcomes. On Kaplan-Meier analyses, the cumulative incidence estimates were significantly different between patients with abnormal regadenoson stress CMRs and those with normal regadenoson stress CMRs (3-year cumulative incidence estimates of 32.1% vs. 12.7%, *p* = 0.034; Fig. 2). Due to the small number of events, multivariable analysis was not performed.

## Discussion

Among heart transplant recipients undergoing regadenoson stress CMR, we found low and similar rates of adverse effects as in non-transplant patients. Importantly, we found no incidence of sinus node dysfunction or high-degree atrioventricular block, including in the first two years after heart transplantation. There were no serious adverse effects. Unlike adenosine, regadenoson appears to be a safe vasodilator stress agent for use after heart transplantation. An abnormal regadenoson stress CMR was associated with a significantly higher incidence of the composite endpoint of major adverse cardiovascular events compared with a normal regadenoson stress CMR.

Our safety findings confirm findings from a prior smaller study of patients undergoing single-photon emission computed tomography (SPECT) stress testing. In 40 recipients at a mean of 9.8 ± 4.5 years after heart transplantation, Cavalcante et al. noted one instance of sinus pause and no atrioventricular block [16]. The same recipients previously had adenosine SPECT and experienced five episodes of second-degree, Mobitz type II atrioventricular block and three episodes of sinus pauses.

Sympathetic reinnervation most often occurs after 18 months and parasympathetic reinnervation most often occurs at around two years after heart transplantation [3]. Since super-sensitivity to adenosine is related to denervation, the exaggerated sinus node and atrioventricular node suppression may be higher early after heart transplantation [17]. With 44% of studies performed in the first two years after heart transplantation, our findings demonstrate, for the first time, that regadenoson is safe also early after heart transplantation when recipients may be more vulnerable to super-sensitivity.

Regadenoson is as efficacious as adenosine for inducing coronary vasodilation [8, 12, 13, 18]. Of the four known adenosine receptor subtypes (A_1_, A_2A_, A_2B_, and A_3_), activation of the A_1_ adenosine receptor accounts for the negative chronotropic and dromotropic effects of adenosine, while A_2A_ is the predominant receptor subtype responsible for coronary blood flow regulation [19]. As a selective A_2A_ receptor agonist, regadenoson has more than 13-fold lower affinity for the A_1_ receptor than for the A_2A_ receptor [20], which explains its lack of super-sensitivity and fewer negative chronotropic and dromotropic effects compared with adenosine.

Our prognostic findings demonstrate, for the first time, the efficacy of regadenoson stress CMRs to identify patients at a higher risk for major adverse cardiovascular events. In many institutions, dobutamine stress echocardiography is used as the non-invasive technique of choice for the routine surveillance of heart transplant recipients. In a large study of 497 consecutive heart transplant recipients, ischemia on dobutamine stress echocardiography was not associated with a composite outcome of death, coronary revascularization, myocardial infarction, and retransplantation [21]. Our findings highlight a potential role for regadenoson stress CMR as a non-invasive modality for the detection of CAV *and* for the risk stratification of heart transplant recipients.

Our study is limited by the single-center, retrospective design, relatively short follow up and a small number of events. We excluded patients with chronic kidney disease (estimated glomerular filtration rate < 30 mL/min/1.73 m^2^). We do not have data on the presence and extent of CAV. However, our study is the first to demonstrate the safety and the prognostic value of regadenoson stress CMR in heart transplant recipients and is the largest study of the safety of regadenoson in these patients. Regardless, we cannot exclude the possibility of adverse effects that occur infrequently (i.e., < 2% incidence). Our findings provide the preliminary data necessary to support a larger, prospective, preferably multi-center, investigation on the utility of regadenoson stress CMR in heart transplant recipients and its comparison with other imaging modalities such as dobutamine stress echocardiography and computed tomography imaging.

## Conclusions

Regadenoson stress CMR is safe and well tolerated in heart transplant recipients, with no incidence of sinus node dysfunction or high-degree atrioventricular block, including in the first two years after heart transplantation. An abnormal regadenoson stress CMR identifies heart transplant recipients at a higher risk for major adverse cardiovascular events.

## Abbreviations

CAV: Coronary allograft vasculopathy
CMR: Cardiovascular magnetic resonance imaging
ECG: Electrocardiogram
IQR: Interquartile range
LGE: Late gadolinium enhancement
LV: Left ventricle/left ventricular
SPECT: Single-photon emission computed tomography
